# Obesity prevention and personal responsibility: the case of front-of-pack food labelling in Australia

**DOI:** 10.1186/1471-2458-10-662

**Published:** 2010-11-02

**Authors:** Roger S Magnusson

**Affiliations:** 1 Faculty of Law, University of Sydney, Sydney, Australia

## Abstract

**Background:**

In Australia, the food industry and public health groups are locked in serious struggle for regulatory influence over the terms of front-of-pack food labelling. Clear, unambiguous labelling of the nutritional content of pre-packaged foods and of standardized food items sold in chain restaurants is consistent with the prevailing philosophy of 'personal responsibility'. An interpretive, front-of-pack labelling scheme has the capacity to encourage healthier patterns of eating, and to be a catalyst for improvements in the nutritional quality of food products through re-formulation. On the other hand, the strength of opposition of the Australian Food and Grocery Council to 'Traffic Light Labelling', and its efforts to promote a non-interpretive, voluntary scheme, invite the interpretation that the food industry is resistant to any reforms that could destabilise current (unhealthy) purchasing patterns and the revenues they represent.

**Discussion:**

This article argues that although policies that aim to educate consumers about the nutritional content of food are welcome, they are only one part of a broader basket of policies that are needed to make progress on obesity prevention and public health nutrition. However, to the extent that food labelling has the capacity to inform and empower consumers to make healthier choices - and to be a catalyst for improving the nutritional quality of commercial recipes - it has an important role to play. Furthermore, given the dietary impact of meals eaten in fast food and franchise restaurants, interpretive labelling requirements should not be restricted to pre-packaged foods.

**Summary:**

Food industry resistance to an interpretive food labelling scheme is an important test for government, and a case study of how self-interest prompts industry to promote weaker, voluntary schemes that pre-empt and undermine progressive public health regulation.

## Background

The food industry and public health groups are locked in a serious struggle for regulatory influence over the terms of front-of-pack food labelling. If the answer to obesity lies in "personal responsibility", as is widely assumed, then one might have assumed that there would be broad consensus over the need for food labelling to unambiguously indicate the benefit of the nutrition in food products offered for sale. Unfortunately, the opposition of the food industry - acting through its peak lobby group, the Australian Food and Grocery Council - to front-of-pack "Traffic Light Labelling" suggests that large food manufacturers and retailers may have something to lose from interpretive labelling schemes that call attention to the quality of the nutrition in terms of salt, sugar and fat content. While such schemes may encourage healthier eating, they may also have a destabilizing impact on purchasing patterns and revenues.

In this article, I examine the role of personal responsibility in public health regulation, and argue that strategies that depend upon motivating individuals to make healthier choices are likely to make only a modest contribution to obesity prevention. This does not mean, however, that improved food labelling has no role to play. Furthermore, its inclusion in the policy package ought to be uncontroversial: ideally, no one should be offended by labelling that improves the capacity of consumers to make healthier choices! While both sides of the debate are keen to argue that their position is supported by research and evidence, I argue that food industry efforts to demonise Traffic Light Labelling and to promote its alternative, "Daily Intake Labelling", are more about protecting industry revenues by heading off government regulation, than empowering consumers to improve their diets.

## Discussion

### (1) Obesity - a growing challenge to health in Australia

Overweight and obesity have become a serious public health challenge in Australia. In 2007-08, 68% of men and 55% of women were either overweight or obese [1], with increased risks for a wide range of chronic diseases including diabetes, heart disease, and several types of cancer [2-5]. Between 1995 and 2004/05, an additional two million Australians became overweight or obese [[6] p 4]. According to one estimate, around one-quarter of diabetes and osteoarthritis, and one-fifth of cardiovascular disease, and colorectal, breast, uterine and kidney cancers are attributable to obesity [[7], p 11]. Overall, in 2003, overweight and obesity accounted for 7.5% of the total burden of disease in Australia, almost as much as smoking (7.8%) [[8] p 74].

Obesity rates are also rising sharply in children. Twenty-five percent of children aged 5-17 are either overweight or obese [1], compared to 21% in 1995, and 12% in 1985 [9]. A recent study by the Victorian Government estimated that if current trends continue, an astonishing 83% of males and 67% of females will be overweight or obese by 2025, as well as one third of children [10].

Evidence from Australia, Britain and the United States indicates that there are discrepancies between measured and self-reported overweight and obesity rates [1,11,12]. The tendency for fewer overweight and obese people to perceive their weight status accurately has important implications if personal responsibility and self-control - based upon an understanding of personal risk factors - are to serve as the key component in strategies for obesity prevention.

Policy options and regulatory interventions available to governments to prevent population weight gain have been canvassed widely in the literature [13-20], and, in Australia, in reports by the Preventative Health Taskforce [21], and the House of Representatives Standing Committee on Health [22]. They include meaningful restrictions on the advertising of high-fat, high-salt and high-sugar foods to children [23], taxes to reduce demand for sugared beverages [24-26], and taxation incentives for private sector employers to invest in health promotion and risk reduction programs for employees [27,28] (with governments also leading by example) [[29] p 142-144]. For all their attachment to individualism and free markets, governments in the United States are experimenting with a wide range of policies for reducing population weight gain [18,19,30], although enormous challenges remain [31].

Central to the blueprint for reform advocated by the National Health and Hospitals Reform Commission was the creation of an Australian National Preventive Health Agency (ANPHA) [32]. Legislation to establish the agency was introduced into Parliament in October 2009, although it has not yet been passed [33]. The functions of the ANPHA would include advising the Health Minister, collecting information, producing reports, and encouraging prevention through partnerships with industry, NGOs and the community [[33], s. 11]. In its final report to the Minister for Health and Ageing, the Preventative Health Taskforce recommended a suite of policies for addressing obesity [21]. These included:

• the establishment of a Prime Minister's Council for Active Living to lead the development of a National Framework for Active Living encompassing the built environment, transport, and social engagement;

• the development of initiatives to encourage and evaluate workplace-based risk modification and health promotion programs ("wellness programs"), and incentives to encourage the uptake of these programs;

• a review of how economic policies and taxation could create incentives or subsidies for improving access to and consumption of healthy foods;

• the establishment of a Healthy Food Compact between governments, NGOs and the food industry to create a healthier and more sustainable food supply; and

• restricting the marketing of energy-dense, nutrient-poor foods before 9.00 pm on free-to-air and pay TV in order to reduce children's exposure (with similar restrictions on premium offers, toys, competitions and promotional characters used to promote these foods).

In addition, the Taskforce recommended the introduction of food labelling on front-of-pack and on menus with "easy to understand information on energy, sugar, fat, saturated fats, salt and trans fats and a standard serve/portion size within three years" [[21] p 13].

The Australian Government has been very selective in its support for the obesity prevention recommendations of the Taskforce. Although agreeing to develop a "soft infrastructure" to support workplace-based prevention programs, it has not supported hard targets for food reformulation, tax-based interventions in encourage healthier food choices, or further restrictions on food marketing to children [34]. On the other hand, the Government is yet to take a position on improved, front-of-pack food labelling. On 23 October 2009, the Australia and New Zealand Food Regulation Ministerial Council (the body responsible for Trans-Tasman food policy), announced the appointment of an independent expert panel, chaired by former Commonwealth Health Minister Dr Neil Blewett, to examine food labelling law and policy in Australia. Amongst other matters, the Blewett Review is to "evaluate current policies, standards and relevant work on health claims and front-of-pack labelling" [35]. The panel will submit its final report late in 2010.

### (2) The impact of "personal responsibility" on obesity prevention policies

The dominant ideology in Australia and many other western democracies is one of commercial freedom and personal responsibility. Assumptions about personal responsibility continue to have a significant impact on policies for the prevention of population weight gain. In well-reported comments in 2006, former British Prime Minister Tony Blair said that:

Our public health problems are not, strictly speaking, public health questions at all. They are questions of individual lifestyle - obesity, smoking, alcohol abuse, diabetes, sexually transmitted disease. These are not epidemics in the epidemiological sense. They are the result of millions of individual decisions, at millions of points in time [36].

Despite framing the problem in this way, Blair went on to emphasise that the role of the state was to "enable" and "empower" individual decisions. This leaves the way open, in Blair's view, for state interventions that empower people to "choose responsibly" [36]. This was consistent with a major paper issued by the Department of Health entitled: *Choosing Health: Making Healthy Choices Easier *[37,38]. In Australia, the emphasis on individual responsibility was well represented by comments made on many occasions by the former Minister for Health and Ageing, Tony Abbott MHR. In 2005, for example, Mr. Abbott stated:

In the end, our weight is largely a product of the amount of exercise we do and the amount of food we put in our mouths. And obviously we are in almost total control of both of those issues [39].

In 2006, in an opinion piece in the *Sydney Morning Herald*, Mr. Abbott wrote:

Unlike cancer or bird flu, there's little mystery about obesity....If the food that people eat contains more calories than they expend in daily living, they put on weight. It's a simple equation....The only way to address habits reinforced by instinct is to tell people what their behaviour is doing to them, over and over again in crystal-clear terms. People need to know that a large Big Mac meal contains 1080 calories, a large chocolate Moove 385 calories, a Krispy Kreme doughnut 198 calories [40].

As dietary advice to individuals, Mr. Abbott's comments make good sense. *For individuals*, taking personal responsibility for one's diet and level of physical activity is the only way (short of bariatric surgery) of achieving energy balance. If our goal is to change population health outcomes, however, it is important to recognise that populations are different from individuals. Firstly, the personal responsibility strategy makes success at the population level dependent on the motivation and capacity of each individual in the population to "live right" and to dramatically alter their habits and lifestyles. As a result, there are as many opportunities for policy failure as there are individuals in the population. Even the weight loss industry - which works with a self-selected group of individuals who *most want to change - *has a rather dismal record in assisting people to reduce their weight in a sustainable manner [41-44]. Most diets fail for most people most of the time. Of course, this does not *necessarily *spell failure in the individual case.

Personal responsibility and personal motivation are unlikely to function as the drivers of mass behaviour change in the absence of an environment that supports and privileges healthier choices. The "problem" is that the average behaviour of the population adapts to, and evolves in response to, the surrounding environment. For example, work and time pressures, urbanization, long commutes, more women in the workforce - and a ready-made food industry that has evolved partly in response to these same factors - mean that the typical Australian meal is very different to what it was fifty years ago. The number of fast food outlets doubled between 1992-2002, with Australian families purchasing fast food "on average once every three or four days" [45]. A substantial body of evidence also demonstrates that certain foods dominate food advertising in a way that is grossly disproportionate to their recommended role in a balanced diet [46]. For example, in 2006, Chapman, Nicholas and Supramanian reported that 31% of their sample of free-to-air television advertisements screened between 7.00 am-9.00 pm were for food, the vast majority of which (81%) were for "foods high in fat, sugar and/or salt, and of low nutritional value" [[47] p 177]. Take-away food, chocolate and confectionary dominated food advertisements, in that order.

Although some individuals may claim that they are immune to food advertising, that's not the point. Food manufacturers and retailers continue to invest enormous sums of money in food advertising because at the population level, it works. According to Scully, Dixon and Wakefield, in 2005 the food industry spent over $110 million promoting their brands in the mainstream media [48]. Globally, in 2004, Pepsico and Coca Cola spent $1.7 and $2.2 billion on advertising, respectively, a total exceeding the World Health Organisation's biennial budget [49].

Until food companies can be persuaded, or are nudged by regulators, to prioritise healthy foods in their promotions, and to improve the nutritional quality of their products across the board, then little is likely to change. The attraction of personal responsibility in political life is not hard to explain. Marketing behavioural change to individuals allows governments to avoid imposing greater responsibility on food, alcohol and tobacco manufacturers and retailers for the health impact of the products they make and sell. Nor does it require governments to improve the quality of the physical environment, or to confront the socioeconomic factors that contribute to health inequalities. Lifestyle recipes are an important change strategy for individuals, and they ought to be encouraged as part of a comprehensive policy response. But they are unlikely to succeed unless governments also tackle the deeper environmental influences that make unhealthy choices and behaviours *common *within the population.

### (3) Confusing lifestyle medicine for population health

The failure to distinguish between policies for improving the health of individuals ("lifestyle medicine") and policies for improving the health of populations ("population health") is central to the attraction of ineffectual policies for the prevention of smoking, obesity, and chronic diseases generally. Advocacy for healthy lifestyle choices in the public sphere (health promotion) is an important strategy in public health practice, but it is best paired with regulation, rather than being seen as an alternative to it, as illustrated by successes in the field of tobacco control [50,51].

Policy-makers should also remember that improvements in behavioural risk factors within a population do not necessarily need to come about through the conscious efforts of individuals. Dental health provides a helpful example. The most effective way to improve the dental health of the population is to put fluoride in the water supply, and to improve access to affordable dental care. In the United States, the fluoridation of drinking water has been assessed as one of the ten most significant public health interventions of the twentieth century [52]. This is not to say that promoting the importance of brushing one's teeth, buying toothpaste, and avoiding sweets should not be part of the policy package. Nor is it to suggest that fluoridation is uncontroversial. Fluoridation of the water supply removes the choice to drink unfluoridated water and to that extent it constrains freedom [53]. Nevertheless, fluoridation has had a silent, beneficial impact on dental health precisely because the success of the policy is not dependent on individual motivation.

Within the context of obesity prevention and public health nutrition, the equivalent of fluoridation is interventions whose effect is to reduce the average number of calories eaten, to reduce the exposure of the population to over-consumed nutrients (salt, sugar, saturated fat), and to increase its exposure to fresh fruit and vegetables. In a liberal, market-based democracy, there are obvious limits to the extent to which these changes in eating patterns can be imposed by legislation [54,55]. Obesity is not contagious. In the context of transmissible disease, the moral justification for coercive regulation depends upon a consensus that government ought to protect the population from health threats to which it has not consented. This is not the case with public health nutrition, especially where regulation seeks to second-guess the dietary preferences of the population. The hard fact remains that improving the national diet partly means taking things away: the salt from the food, the saturated fat from the diet, the snacking from the lifestyle. This is qualitatively different from the public health challenges of the past century, which involved providing more of what was absent, whether it be sanitation, clean water, immunization or health care services [56].

A final point to make about the over-emphasis on "personal responsibility" as a strategy for reversing population weight gain is that it is likely to fail most spectacularly among those who currently suffer the greatest health inequalities, relative to the rest of the population, due to the fact that their health status is undermined by socio-economic disadvantage [57,58]. In Australia, rates of obesity have risen for all socioeconomic groups, but a socioeconomic gradient remains and is particularly pronounced when obesity is compared with level of education, income quintile, and occupation [59]. One plausible interpretation of the expansion of fast food restaurants during the 2008-2009 global economic downturn is that people with limited spending power, and people who are living with economic insecurity, are more likely to purchase the cheaper, energy-dense meals that these restaurants provide [[31], pp 65-67] [60,61]. Similarly, the cheaper cost of energy-dense, less nutritious food, relative to energy-dilute foods, including fresh fruit and vegetables, may be one factor contributing to disparities in diet, and thus to the social gradient of obesity [62,63]. To the extent that it enables consumers to identify healthier foods, effective food labelling may facilitate a healthier diet. The reality, however, is that improved food labelling is likely to be least effective in encouraging healthier choices among those with the greatest levels of social and economic disadvantage.

### (4) The population health strategy

To summarise so far: to make progress on obesity, governments need to focus on policies that could influence the average behavior of the population; not necessarily dramatically (dramatic changes, like dramatic diets, tend not to be sustainable), but subtly, through small, sustainable modifications to the behavior of many people. Public health advocates often speak about creating supportive environments that encourage healthy choices and lifestyles. By changing the environment, and making healthy lifestyles easier, it becomes "less necessary to keep on persuading individuals" [[64] p 431].

Of course, a population health strategy has its own challenges. To improve the weight and health of the population, we need to change eating patterns, the nutritional quality of the food eaten and levels of physical activity. This involves reducing the consumption of high-sugar, high-fat, high-salt foods, and increasing the consumption of fresh fruit and vegetables. This can lead public health policy-makers into direct conflict with those who benefit from the current, patterns of (over)consumption that fuel chronic disease.

One of the constraints on the capacity of the food industry to contribute to improved nutrition is the potential for misalignment between the economic incentives that drive industry behaviour and improved public health outcomes. For example, "'good' foods are [frequently] bad commodities with low profit margins while 'bad' foods are good commodities with high margins" [[65] p 1247]. This is not to say that the food industry is unwilling to meet demand for healthy foods, or to trial healthier foods. The participation of the food industry is vital, moreover, when it comes to portion control, increasing healthy nutrients, reducing over-consumed nutrients (salt, sugar, saturated fats), and affordability. Notwithstanding the success of bans on trans fats [66], these things are difficult, at least in political terms, to legislate. At the same time, there are powerful disincentives for a rational, economically-motivated actor like a food or beverage manufacturer to do anything that could destabilize the revenues that flow from the current demand for unhealthy foods.

### (5) Food labelling and public health nutrition: a case study

Food labelling provides an interesting case study of the contest around food regulation, and the way in which this obliges the food and beverage industry to protect its economic interests by participating in the debate about obesity prevention and public health nutrition. Given the dominance of the "personal responsibility" narrative in public life, one might have thought that labelling laws designed to enhance the capacity of adults to make informed dietary choices for themselves and their children would have sailed through Parliament long ago. The reality is rather different.

On the one hand, the food industry has a strong economic interest in being able to develop and advertise the health benefits of "functional foods". In Australia, a new draft Standard on Nutrition, Health and Related Claims (Standard 1.2.7), and amendments to the Nutrition Information Requirements set out in Standard 1.2.8 of the Food Standards Code, provide the framework that supports food industry initiatives in this area [67]. To the extent that it can make claims about the health effects of certain nutrients in food, the food industry seems to have embraced the notion of "healthy foods". On the other hand, when it comes to junk foods, the industry prefers to talk about healthy diets and healthy choices.

The Food Standards Code, which applies in each State and Territory, requires packaged foods to display a nutrition information panel indicating the amount of energy, protein, total and saturated fat, carbohydrate, sugars and sodium (salt) per recommended serving and per 100 g or 100 ml [68]. "Truth in nutrient claims" is required. For example, "reduced fat" foods must not contain more than 75% of the fat content of the industry norm for that particular type of food; there must also be at least a 3% reduction per weight for food, and 1.5% per weight for liquid food. "Low fat" foods must not contain more than 3% fat per weight, and "fat free" foods must not contain more than 0.15% fat per weight [69].

Studies suggest that Australian and New Zealand consumers find current back-of-pack labelling confusing and difficult to understand [70]. Similarly, in their review of international studies, Cowburn and Stockley point to the difficulty consumers have in understanding the significance of nutrient information within the context of their overall diet [71]. They argue that interpretational aids or benchmarks, such as verbal descriptors or guideline daily amounts, could assist consumers to place a product "into a total diet context" [71].

In November 2006, one month after front-of-pack food labelling reached the agenda of the Australia New Zealand Food Regulation Ministerial Council, the Australian Food and Grocery Council (AFGC) launched a voluntary labelling scheme called "Daily Intake Labelling". Daily Intake Labelling is currently used by around 180 brands and is backed by Woolworths, Coles, Franklins, the Australian Beverages Council and the Confectionary Manufacturers of Australia [72]. It identifies the percentage of energy, protein, fat, saturated fat, carbohydrates, sugars and sodium *per serve *of the food in a thumbnail, monochrome format. To this extent, it replicates on the front of the pack information that the Food Standards Code requires manufacturers to include in the nutrition panel.

The main contender to Daily Intake Labelling is called "Traffic Light Labelling". Traffic Lights *judge the nutritional quality of food *by means of highly visible red, amber and green flags on the front of the pack, together with percentages that reflect the levels of salt, sugar, fat and saturated fat in the food [73]. In 2005, the UK Food Standards Agency evaluated both Traffic Lights and Daily Intake Labelling (referred to as Guideline Daily Amounts, or GDAs), and found that Traffic Lights were most likely to meet the objectives of supporting healthier decisions and eating ([74], p 9). Although both approaches remain voluntary in the United Kingdom, a large number of public health associations have come out in support for Traffic Lights [75,76]. On 22 June 2010, the National Institute for Health and Clinical Excellence (NICE) issued a report which included a call for Traffic Light Labelling to become the national standard [77]. The Food and Drink Federation issued a news article on the same day, attacking NICE and claiming that 93 manufacturers and retailers have now embraced front-of-pack labeling based on Guideline Daily Amounts [78].

In June 2009, the Food Standards Agency published independent research recommending a single front-of-pack label incorporating elements of both schemes, including traffic light colours, the words "low", "medium" and "high", the levels of nutrient in the product, and the percentage of recommended Daily Intake (Guideline Daily Amounts) [79]. However, the Coalition Government in the United Kingdom does not support regulation in this area, and in England, the role of the Food Standards Agency in non-safety-related food labeling has been transferred to the Department for Environment, Food and Rural Affairs [80].

In Australia, research conducted by a coalition of Australian organizations including CHOICE and the Cancer Council [81], support the conclusion that overall, Traffic Lights are the most successful model for helping consumers to identify the foods that contribute to a healthier diet. In the latter case, a survey of 790 adults in New South Wales found that consumers using traffic light labelling were "five times more likely to correctly identify the healthier food products, compared to the Monochrome %DI [percentage of daily intake] system" ([81], p 15). As a spokesperson for CHOICE states, "Traffic lights are simple and easy to use. They enable busy consumers to make healthy choices while doing their shopping. Shoppers don't have all day to stand around in supermarket aisles calculating their dietary needs" [82].

In February 2009, a Consensus Forum hosted by the Australian Chronic Disease Prevention Alliance agreed that any front-of-pack labelling scheme ought to be mandatory, interpretive, and should convey nutrient information based on 100 ml or 100 g, rather than based on a serving size determined by the manufacturer [83]. The Alliance also recommended extending this form of nutrition labelling to the standardized menus of quick serve restaurants.

There are two critical differences between Daily Intake Labelling and Traffic Light Labelling. Firstly, Daily Intake Labelling is more complex than Traffic Lights because the former requires comparison across seven categories. It also assumes that consumers measure out and limit themselves to the recommended serving. This is unrealistic: when did *you *last measure out 30 grams of cereal at breakfast? Furthermore, in order to use Daily Intake Labelling as an effective aid to a healthier diet, consumers must compare nutrients from different serving sizes of the similar products the consumer is choosing between, since serving sizes vary and are the responsibility of each manufacturer [84]. Further, consumers must keep a tally of the foods consumed during the day in order not to over-consume "negative" nutrients such as fat, and in order to achieve daily targets for "positive" nutrients such as fibre. They must also consider how their individual daily intake needs compare with those of an average adult male ([85], p 124).

Secondly, unlike Traffic Light Labelling, Daily Intake Labelling is agnostic about the quality of the *nutrition *of a product. This explains why the Sanitarium Health Food Company has not signed on to Daily Intake Labelling, stating that: "The % daily intake value only provides information about the quantity of nutrients not the quality of nutrition" [86].

Traffic Light Labelling is *interpretive and judgmental*. It helps consumers to make healthier choices by taking a position on the nutritional content of the product. It identifies the foods you should avoid or eat sparingly! It is this judgmental quality of Traffic Light Labelling, together with its relative simplicity, that makes it more helpful for making decisions in real-time, in the aisles of supermarkets and corner stores. With certain refinements, the interpretive function of Traffic Light Labels can be further enhanced in ways that respond to common criticisms. For example, as recommended by the UK Food Standards Agency, red lights can be reserved for added sugars, together with clarifying statements such as "This product contains naturally occurring sugars" [87]. This relatively simple variation would enable consumers to distinguish "between cereals which are high in added sugars, and those high in sugars due to high fruit content" ([87], p 5). It may also make sense to vary the cut-off points for certain foods such as cheese, which is naturally high in fat, and where the Traffic Lights would otherwise fail to distinguish between full fat and reduced fat variants (both would attract a red light) [85].

Although Traffic Light Labelling may assist consumers to readily identify products with high levels of fat, salt and sugar, there are many links in the chain between the introduction of such a scheme and dietary improvements at the population level. Sacks, Rayner and Swinburn found that the introduction of traffic light labelling by a major retailer on a selection of sandwich and ready-meal lines had no impact on the relative healthiness of consumer purchases in the four weeks after introduction [88]. They argue, however, that it is possible that Traffic Light Labelling could impact consumer purchasing patterns in the longer term, or if labelling covered a wider range of products. They highlight the potential for traffic lights to drive product reformulation (by manufacturers keen to avoid red lights), and the impact of labelling on consumer awareness of what they are eating, regardless of its direct impact on purchasing behaviour. More broadly, although there is evidence that consumers are interested in nutrition labelling [89], there is less evidence that consumers use it [90]. While these cautions apply to both Traffic Lights and to Daily Intake Labels, the relative simplicity of Traffic Lights suggests that it may be easier to influence consumer behaviour over the longer term using this system.

Perhaps the most interesting challenge to Traffic Light Labelling, when compared to Daily Intake Labelling, comes from the possible "health halo effect" of products bearing amber and green lights. In their study of low-fat labels, Wansink and Chandon found that while low-fat labels increased consumption overall, it had a dramatic effect on the amount consumed by overweight consumers - "the very people for whom calorie underestimation is most harmful" [91]. The authors argue that this effect may be explained by the way in which low-fat labels change perceptions about the calorie density of the food, about the appropriate serving size, as well as the level of guilt associated with consumption. It is not clear that any "health halo" associated with low-fat claims would transfer to products low in salt, sugar or fat when all products were routinely labeled using Traffic Lights. If, however, it could be shown that Traffic Light Labelling led consumers to consume more salt, fat, sugar or calories than they otherwise would, thereby undermining the intended impact of these labels, it would represent a challenge to the capacity of interpretive labelling schemes to assist consumers to improve their diet and to exercise personal responsibility [92].

To the extent that Traffic Light Labelling has the capacity to assist consumers to moderate their consumption of red-flagged foods, it also has the capacity to interfere with the revenues of food manufacturers and retailers. This may explain some of the antipathy of the AFGC to Traffic Lights, and why it has been vigorously promoting its non-interpretive alternative - Daily Intake Labelling [93]. It is worth emphasising, however, that the possible benefits of Traffic Light Labelling are not only mediated through their impact on the purchasing behaviour of consumers, but through their capacity to encourage "food manufacturers to change the food supply through product reformulation and development to meet nutrient criteria levels" [[94], pp 24, 25]. In some cases, it may not be possible for manufacturers to shift from red to amber lights without sacrificing features that are essential to consumer appeal (for example, it may be difficult to reduce salt levels in potato chips to the level required to avoid a red light). In these cases, there may be little incentive for product reformulation, and manufacturers are likely to resent any obligation to brand their product with a red light.

The Chief Executive of the AFGC has argued that Traffic Light labelling "is an overly simplistic approach to the very important issue of food labelling in Australia" [95], and has welcomed the Commonwealth government's recent decision to defer any decision on the preferred model for 12 months [96]. If the AFGC is committed to improving consumers' understanding of the nutritional profile of foods available for sale, in order to facilitate healthier choices, then it is difficult to understand why the deferral of a decision about front-of-pack labelling regulation should be welcomed. The AFGC's position only makes sense once it is realized that delaying any decision will enable the AFGC to claim that its own already-implemented, non-interpretive scheme has become the industry standard and that any further change would be unnecessary, confusing and disruptive. Voluntary, industry sponsored initiatives are a familiar feature of the food governance landscape, both in Australia, and internationally. Their functions include heading off legislation, protecting revenues, and "positioning" the industry so that it is seen as responsive to concerns about diet and nutrition [97].

### (6) Beyond pre-packaged foods: Personal responsibility and food labelling in chain restaurants and franchises

In the United States and, more recently, in Australia, the debate about nutrition labelling has moved beyond pre-packaged foods to the standardized food items regularly offered for sale in chain restaurants [98]. In the United States, over the forty years to 2002, the percentage of the total food budget spent on food prepared away from home increased from 27 to 46 percent [99]. The average American eats 218 restaurant meals per year [100], and this accounts for around one third of calories consumed [99]. In Australia, there are around 16,000 fast food outlets serving 1.6 billion meals a year [101].

Studies suggest that consumers persistently under-estimate the calorie content of restaurant meals. In one study, Burton and colleagues found that the real calorie values of a group of "less-healthful" food items were nearly twice as high as consumer estimates [102]. Consumers under-estimated levels of fat and saturated fat in less-healthful items by amounts exceeding 60% of the recommended daily values. Consumers also under-estimated salt levels by 341% [102,103]. These discrepancies provide a powerful argument for extending nutritional labelling requirements to the standardized menu items that are offered for sale in chain restaurants and franchises [104]. Restaurant calorie labelling initiatives have the capacity to improve the diet of the population through two important pathways: by informing consumers of the nutritional profile of foods in ways that lead to healthier choices, and perhaps more significantly, by providing a catalyst for the re-formulation and improvement of menu options.

In September 2008, California became the first U.S. State to require menu labelling of standard items offered for sale by restaurant chains and franchised establishments with at least fifteen outlets [105]. Each restaurant with a standard menu is required to indicate, beside each menu item, the total number of calories, grams of saturated and trans fat, and the total number of carbohydrates and milligrams of salt [105]. A similar law took effect in Philadelphia from 1 January 2010 [106]. More limited legislation, which requires the display of calorie information in chain restaurants, operates in four States, and several cities, including New York City [31,107,108]. In Massachusetts, the law applies to restaurant chains with twenty or more outlets, and also to drive-through lanes [109].

These State and city initiatives have been significantly impacted by President Obama's health care reform legislation, the *Patient Protection and Affordable Care Act *(H.R. 3590). This Act contains nutrition labelling provisions that require calorie counts to be placed on the menu for standard items appearing on the menu for at least 60 days per year, in retail food establishments with 20 or more locations [110]. Calorie disclosures must also be accompanied by a statement of the recommended daily caloric intake, in order to educate consumers of the significance of the calorie information in the context of a total daily diet. Calorie disclosure provisions also apply to drive-through menus, vending machines, and self-service food. Importantly, the Act allows food establishments not caught by the provision to "opt in", thereby preempting state and city legislation which, like the California Act, may go beyond calorie counts to require nutrient disclosures [111].

In Australia, the Victorian government has foreshadowed an initiative that would require food businesses with 50 outlets in Victoria, or more than 200 outlets nationally, to display calorie information on menus and menu displays from the second half of 2012 [112]. The New South Wales Government, by contrast, in its submission to the Blewett Review, has suggested that fast food chains should disclose the calories, saturated and trans fat, and salt content of menu items [113]. This is consistent with recommendations by the Heart Foundation, which has also called for a mandatory national scheme that would apply to cafes, bakery and other quick service stores, ice-cream and juice bars with 20 or more outlets [101].

There is an emerging body of evidence that calorie disclosures do influence the number of calories purchased and that calorie labelling could have a moderating impact on over-eating at the population level [114-117]. In a study that compared purchasing patterns in restaurants in low-income neighbourhoods in New York City (calorie labelling) and Newark (no calorie labelling), Elbel and colleagues found no evidence that "menu labelling influenced the total number of calories purchased at the population level" [118]. One hypothesis that deserves consideration is that nutrition labelling may have a commensurately greater impact in moderating calorie intake and increasing the consumption of healthier options within middle-income neighbourhoods, rather than in low-income areas where the label that matters most is the price, and where economic pressures drive consumers towards more energy-dense options [119]. This effect, if found, would be consistent with the social gradient of chronic disease and its risk factors, and with the argument suggested above that the personal responsibility strategy is likely to be least successful among those who are most disadvantaged. This calls attention to the fact that while nutrition labelling is important, it is no panacea. Nevertheless, restaurant menu labelling requirements should be introduced as part of the wide-ranging basket of policies that will be needed if we are to improve the food and physical activity environments [18,21,120-122].

## Summary

Front-of-pack labelling provides an important test of the ability of the food industry to convince government that there are no unhealthy foods, only unhealthy diets - for which individuals are alone responsible. If governments are unable to resist industry opposition to providing consumers with nutritional information in an effective format in order to support healthier choices, then prospects for introducing other measures to combat rising obesity rates do not look positive. Traffic Light Labelling is, after all, aligned with the personal responsibility ethos. It is not coercive, it is not patronizing. It does not mandate what food products the food industry can manufacture, nor what adults can or cannot eat.

Unlike the food industry's voluntary scheme, however, Traffic Light Labelling does make judgments about the quality of the nutrition provided by different foods. This is precisely what makes it useful to consumers. Furthermore, Traffic Light Labelling is likely to be much simpler for consumers to use. It does not require comparison across seven categories, nor is it based upon a "recommended serving" size that consumers, on average, are likely to ignore or misunderstand. Finally, if Traffic Light Labelling is introduced, consumers will not be denied the information that the industry has re-packaged as Daily Intake Labelling: such information is already available on the back of the pack. What Traffic Lights provide is a tool for making healthier decisions, rapidly, and in real time. For these reasons, the Australia New Zealand Food Regulation Ministerial Council ought to make Traffic Light Labelling a mandatory standard for pre-packaged foods in Australia and New Zealand. While this does not necessarily mean that Daily Intake labels, as advocated by the AFGC, should be removed, governments should certainly resist food industry pressure to eliminate interpretive front-of-pack labelling.

Meals eaten out in "fast food" or chain restaurants are a fact of life in Australia and New Zealand. The inability of consumers to accurately assess the energy values or levels of fat, saturated fat, sugar and salt in these meals provides a compelling reason for extending nutritional labelling beyond supermarkets. At the present time, consumers can only guess, and calls for more personal responsibility are unlikely to result in individuals abandoning fast food restaurants en masse in favour of home-cooked meals! If the obesity epidemic is framed as a problem for individuals who must exercise personal responsibility, what possible justification could there be for denying consumers access to nutritional information in a highly visible format, for all standardized menu items? For the same reasons that Traffic Light Labels could encourage healthier choices in supermarkets, they could also lead to healthier patterns of consumption in chain restaurants and franchises. More importantly, interpretive nutrition labelling is likely to act as a stimulus for food companies to improve the nutrition of their standardized offerings, creating a virtuous cycle.

## Competing interests

The author declares that they have no competing interests.

## Pre-publication history

The pre-publication history for this paper can be accessed here:

http://www.biomedcentral.com/1471-2458/10/662/prepub
